# Turns with multiple and single head cast mediate *Drosophila* larval light avoidance

**DOI:** 10.1371/journal.pone.0181193

**Published:** 2017-07-11

**Authors:** Weiqiao Zhao, Caixia Gong, Zhenhuan Ouyang, Pengfei Wang, Jie Wang, Peipei Zhou, Nenggan Zheng, Zhefeng Gong

**Affiliations:** 1 Department of Neurobiology, Key Laboratory of Medical Neurobiology of the Ministry of Health of China, Key Laboratory of Neurobiology, Zhejiang University School of Medicine, Hangzhou, Zhejiang, China; 2 Qiushi Academy for Advanced Studies, Zhejiang University, Hangzhou, Zhejiang, China; Biocenter, Universität Würzburg, GERMANY

## Abstract

*Drosophila* larvae exhibit klinotaxis when placed in a gradient of temperature, chemicals, or light. The larva samples environmental stimuli by casting its head from side to side. By comparing the results of two consecutive samples, it decides the direction of movement, appearing as a turn proceeded by one or more head casts. Here by analyzing larval behavior in a light-spot-based phototaxis assay, we showed that, in addition to turns with a single cast (1-cast), turns with multiple head casts (n-cast) helped to improve the success of light avoidance. Upon entering the light spot, the probability of escape from light after the first head cast was only ~30%. As the number of head casts increased, the chance of successful light avoidance increased and the overall chance of escaping from light increased to >70%. The amplitudes of first head casts that failed in light avoidance were significantly smaller in n-cast turns than those in 1-cast events, indicating that n-cast turns might be planned before completion of the first head cast. In n-casts, the amplitude of the second head cast was generally larger than that of the first head cast, suggesting that larvae tried harder in later attempts to improve the efficacy of light avoidance. We propose that both 1-cast turns and n-cast turns contribute to successful larval light avoidance, and both can be initiated at the first head cast.

## Introduction

Phototaxis is a behavior that animal travels between environments with different light conditions. It could be crucial for animal survival. *Drosophila* larval phototaxis has received much investigation in the past 50 years [1,2]. In recent years, much progress has been made in elucidating the neural basis of larval phototaxis [3,4,5,6,7,8]. Neurons extending from the primary visual sensory neurons of Bolwig organs (BOs) to secondary neurons such as PDF neurons and fifth lateral neurons [9,10,11], and to neurons such as prothoracicotropic hormone (PTTH) expressing neurons, have been identified [4,7]. A number of behavioral assays have been used in studying larval phototaxis, including a light/dark assay in which an agar plate is divided into two halves with one half shaded. A group of larvae is allowed to crawl on the plate and choose between the dark and light regions, and a performance score is calculated by counting the number of larvae on each side. In another assay, a larva crosses light/dark boundaries on a checkerboard test. In this case, the light/dark choice is encountered frequently [8, 12]. In one other assay, light is shed on crawling larvae and their responses to on/off switch of light are monitored and recorded [8, 13, 14, 15]. In a fourth assay, a small light spot is directed onto the head of a crawling larva to observe its behavioral response [5].

Animal avoidance generally uses a strategy of klinotaxis or tropotaxis. In klinotaxis the animal uses temporal comparison of sensory information to determine the direction in which to proceed. In tropotaxis, spatial comparison of the inputs to the left and right sensory organs determines the direction of movement. *Drosophila* larvae primarily adopt a klinotactic strategy in relation to thermotaxis [16, 17] and chemotaxis [18, 19]. Larvae show more turning when heading against a preferred stimulus gradient and less frequent turning when heading along the favored gradient. In *Drosophila* larva, avoidance is realized mainly through large-size turning, although small-size directional bias may also be utilized[20]. Turning generally includes two steps: first casting the head to choose a new direction, and then straightening the body to align with the new direction [21]. Neurons commanding turning behavior are thought to be located in the region of the subesophageal ganglion in the larval central nervous system [22]. Activation of sensory neurons responsive to chemical repellents might elicit the turning response by activating turning-command neurons [23]. Before the larva makes a turn, it may cast its head just once, or it may exhibit a sequence of several head casts. The direction of headcast usually switches alternately to the opposite as larva continues to cast its head. This change in head cast direction is usually considered as a rejection of the result of the previous cast, which failed to bring the larval head (or sensory organs) into a more favorable position. Thus, whether the larva initiates another head cast is determined by the result of the previous head cast [8,16,17].

Here, we developed a light-spot assay to investigate larval phototactic behavioral responses to light stimulation within a short period, including before, during and after entering light. In this assay, the behavioral details of the responses of an individual larva to the dark-to-light transition were recorded and analyzed. We focused on larval head-cast behavior. We observed that a larva may cast its head once (1-cast) or several times (n-cast), apparently exploring the environmental conditions before making a turn to escape from light. Although less commonly observed than 1-cast turns, n-cast turns greatly improved larval performance in light avoidance. Unexpectedly, we found that n-cast turns could be initiated independent of 1-cast turns, that is to say, n-cast turns were not simply a result of rejection of the first head cast that failed in light avoidance. In n-cast turns, the amplitude of head casts varied, with the second cast larger than the first. We discuss the significance of the multiple-head-cast strategy in larval avoidance behavior.

## Materials and methods

### Fly culture

*w*^*1118*^ flies were raised at 25°C on standard medium in a 12:12 h light: dark cycle. Third instar larvae of 72–96 hours after egg laying were used.

### Light spot assay

The setup, placed in a dark room, consisted of: an LED (Thorlabs Inc. Newton, NJ, USA) for blue light illumination; a web camera with an 850±30 nm band-pass infrared filter in front of the lens to allow infrared imaging; LED generating infrared light placed aside the test arena; an 1% agar plate (1mm in thickness) as arena for behavior test (S1 Fig). In behavioral experiments, a beam of blue light (460 nm) was projected through a round hole in a foil sheet to form a 2-cm diameter light spot on an agar plate. The light intensity was 0.44 μw/cm^2^ in the light spot (S1 Fig).Light intensity changed for ~40 folds within 2-3mm at the rim of the light spot (S2 Fig).

Before each behavioral test, an individual third-instar larva (72–96 h after egg-laying) was rinsed with distilled water and transferred to the agar plate with the light spot positioned at the center. The larva was allowed to adapt to the new environment for 2 min. Immediately before the start of the phototaxis test, the larva were moved to a position 2–4 cm from the light spot and reoriented toward the spot. The larva then crawled toward the light spot and either entered or avoided the light. The whole process (usually less than 1 minute) was recorded with the web camera at a frame rate of 60 fps until the larva moved away from the light spot. The video was then analyzed with Matlab (Mathworks Inc. Natic, MA, USA)-based SOS (Sensory Orientation Software) software (https://sourceforge.net/projects/sos-track/) and custom scripts[24] (S3 Fig, see ref. [24] for comparison of flowcharts, and S4 Fig). 1 mm corresponds to 11.94 pixels in length in all videos.

### Data analysis and behavioral definitions

Videos of larval phototaxis were batch processed with SOS as reported previously [24] to store the following raw data in a file (motorData.m): frame rate; larval image contour; skeleton; positions and speed of the head, midpoint, tail and centroid; headtheta; headomega; bodytheta; and bodyomega. The variables headspeed, midspeed, tailspeed and cmspeed were the velocity of head, midpoint, tail and centroid. The variable headtheta is the angle between the head-midpoint axis and the midpoint-tail axis; headomega is the angular speed of headtheta, which is a measure of head-cast speed; bodytheta is the angle between the midpoint-tail axis and the horizontal edge of the arena; bodyomega is the angular speed of bodytheta, which measures the turning speed. Image processing of light spot was realized with custom written script (lightarea.m).The light spot data was stored in a file named lightregion.m.

Phototaxis related head cast behavior was processed with matlab-based custom written scripts (turn_classify.m with cast_max_frames.m and peakdet.m adopted from http://www.billauer.co.il/peakdet.html), in which the light spot edge was smoothed with a Sgolayfilter with polynomial order of 12. Lines of headomega, bodyomega and tailspeed were smoothed using a moving average filter. The light induced head cast related data were stored in a file named light_turn_event.m. To quantify head cast behaviors, a stop period was defined as occurring when tailspeed was lower than 0.21mm/s (2.5 pixels/s, this and the thresholds hereafter were arbitrarily set according to experience). Short intervals between sub-threshold periods were ignored as they were considered to belong to a continuous stop period. Similarly, very short periods of sub-threshold tailspeed were ignored. In stop period, a head cast event was considered to occur when the headomega peak value exceeded20.05°/s (0.35 rad/s). A head cast ended when headomega following the peak dropped to zero. A head cast was defined as accepted if there was no further head cast during the same stop period. A head cast was defined as rejected if there was another head cast that followed in the same stop period.

**Fig 1:**
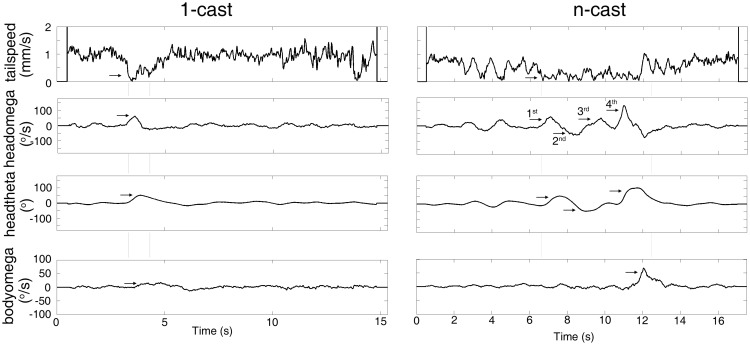
Typical 1-cast and n-cast turns in light-spot based phototaxis. The tailspeed (top row), headomega (second row), headtheta (third row) and bodyomega (bottom row) time series of a larval 1-cast (left column) and a 4-cast (right column) turning events. Rectangular frames show the stop periods. Arrows in tailspeed point to the onset of the stop period. Arrows in headomega point to headomega peaks indicating head casts following stop. Arrows in headtheta point to the largest body angles. Arrows in bodyomega point to the bodyomega peaks which indicate larval turning. In 1-cast, the larva entered the light spot at 2.87 second. In 4-cast, the larva entered the light spot at 2.0 second, the stop period indicated by the rectangular frame is the second stop period after larva entered light spot.

A turning behavior with head cast(s) was identified as following: first a drop in tailspeed to sub-threshold level, marking the beginning of a stop period; then an above-threshold headomega peak(s) that marked the head cast(s); and finally, an above-threshold bodyomega peak that marked the turning of the midpoint-tail axis. As turning behavior often involved straightening of larval body and produced headomega peaks in absence of head cast, such headomega peaks were excluded when defining head cast behavior. In a 1-cast turn, as shown in Fig 1 (left column), there was a single headomega peak between the onset of the stop period, which was marked by the point at which tailspeed dropped to below 0.21 mm/s, and the peak of bodyomega, which indicated the turning behavior. For an n-cast turn, there are at least 2 headomega peaks between the start of stop and turning indicated by a bodyomega peak (right column in Fig 1, S1 Video, S2 Video).

For investigation of larval light avoidance behavior, only turning events that potentially resulted from light avoidance were considered. The distance of the larva from the light spot was defined as the distance between larval head and nearest point on spot circumference. Distances were defined as positive when the larval head was outside the light spot, and negative when it was inside the light spot. For turning events initiated out of light spot, only those happened within in 20 pixels (about 2mm) to the edge of light spot were considered as light avoidance related. For turning events initiated inside light spot, only those happened within 50 pixels (about 4mm, i.e. the length of larva, a range that could be covered by a large size head cast) to the edge of light spot were considered as light avoidance related. Furthermore, turning events after 5 seconds of total light exposure time were not considered to exclude possible light adaptation effect. In these light avoidance related turning events, a head cast was defined as successful in light avoidance when the head was out of light by the end of the head cast (the time that the headomega peak dropped to zero); a head cast was defined to fail in light avoidance if larval head remained in the light spot by the end of the head cast. As larva might detect light out of the light region defined by computer due to light scattering effect, the edge of light spot was expanded outward for 10 pixels (about 0.84 mm) when measuring success of light avoidance.

The scripts are available at https://github.com/oyzh/Larva-Tracking.

All raw data in form of videos are available at following links:

https://figshare.com/articles/6_17-6_18/5117392

https://figshare.com/articles/0719/5117395

https://figshare.com/articles/0413/5117404

https://figshare.com/articles/0718/5117413

**Statistics**: The binomial Fisher’s exact test was used to compare success rates and acceptance/rejection ratios. Student’s *t*-test was used for comparison of headomega peak values.

## Results

### Head cast identified in light-spot-based phototaxis

We used a light-spot assay to characterize larval movement during phototaxis. In this assay, an LED-generated spot of blue light was projected onto an agar plate in a dark environment (S1 Fig). Single larvae, initially adapted to the dark region for 2min were allowed to run into the light spot and to escape (S4 Fig, S1 Video).

We focused on the turning behavior of larva because turning is the most important aspect of avoidance behavior.

As shown in Fig 1, a 1-cast turn included a single headomega peak between the onset of the stop period and turning(left column of Fig 1),whereas there were at least 2 headomega peaks between the start of stop period and turning in n-cast turn (right column in Fig 1).

### n-cast turns improve larval light avoidance

**Fig 2:**
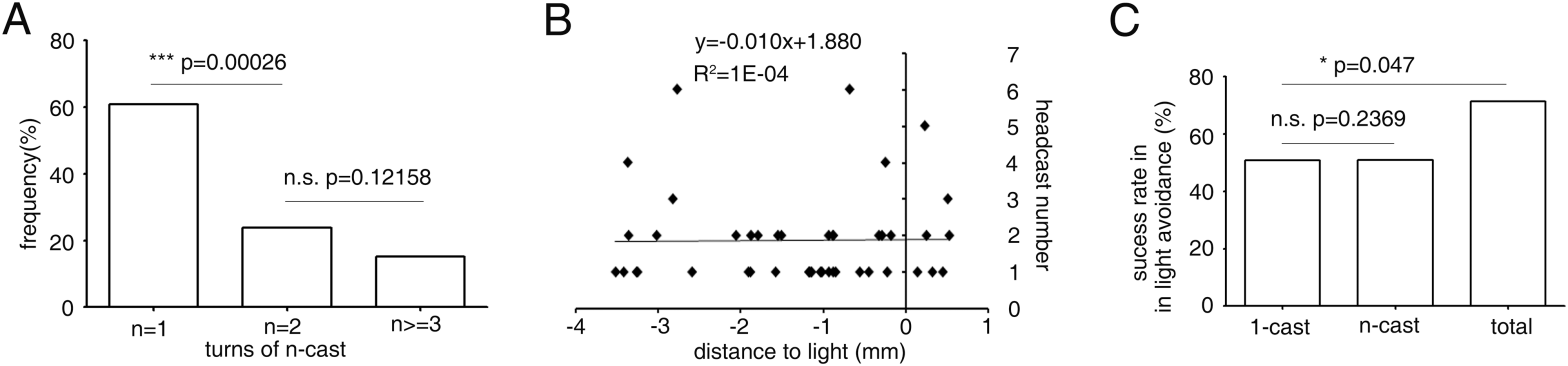
Properties of 1-cast and n-cast turns in larval fast phototaxis. (A) Frequency of turns with one head cast, two head casts, and three or more head casts. The frequency decreased as the number of head casts increased. n = 46. (B) The distribution of turns with different numbers of head casts in relation to the distance from the edge of the light spot at the time that turning was initiated. There was no obvious correlation between the distance and number of head casts. Positive distance means out of light spot, negative distance means inside light spot. n = 45. (C) The probability of successful light avoidance after 1-cast and n-cast turns was lower than the overall probability of successful light avoidance. n = 28, 18and 35 respectively for 1-cast, n-cast and total. **, *P*<0.01, ***, *P*<0.001, Fisher’s exact test.

We noticed that 1-cast turns and n-cast turns occurred before and after the larva entered the light spot, respectively. We speculated that 1-cast and n-cast turns might play different roles in avoidance behavior. We focused on the light-evoked turns that occurred within the range of the light spot (see Methods). Turns with only a single head cast were most common; about 60.87% of all turns were 1-casts. Multiple head-cast turns of two or more occupied, respectively, 23.91% and 15.22% of all turns (Fig 2A). We combined all head-cast turns of two or more casts as n-cast turns, and compared 1-cast turns with n-cast turns later on. We also investigated the location of single and multiple head-cast turns, which was defined as the position where a stop period started. 1-casts and n-casts appeared to occur with approximately equal probability at a range of distances (positive and negative) from the edge of the light field (Fig 2B). This suggested that the experience of light exposure did not affect the number of head casts before turning. Surprisingly, no obvious correlation was seen between the magnitude of head casts and the larval orientation angle with respect to the light spot, for both the first and second head casts (S5 Fig). One possible reason is that the larval orientation angles were mostly within the range of 40-90°, in which corresponding head cast size probably did not differ much. Another possible reason is that the light gradient at the edge of light spot was steeper than in a previous report so that the effect of the orientation angle on the head cast was overcomed[8]

Since turning is crucial for light avoidance, we next looked at the success rate of 1-cast turns and n-cast turns. A successful turn was one that brought the larval head out of the light region by the end of the turn. Success or failure of head casts was similarly defined. The success rate of 1-cast turns was about 50.0%. To our surprise, the success rate of n-cast turns was 50.0%, which was the same as that of 1-cast turn (Fig 2C). The overall success rate of turns, including both 1-casts and n-casts, was 71.43%, which was significantly higher than that of 1-cast turn. The overall success rate was not a simple addition of the success rates of 1-cast and n-cast because one larva might perform 1-cast and n-cast turns sequentially when attempting to avoid light. This data suggested that n-cast turns contributed much to success in light avoidance, although more than half of all turns were 1-cast turns.

### n-casts are planned before the first head cast

**Fig 3:**
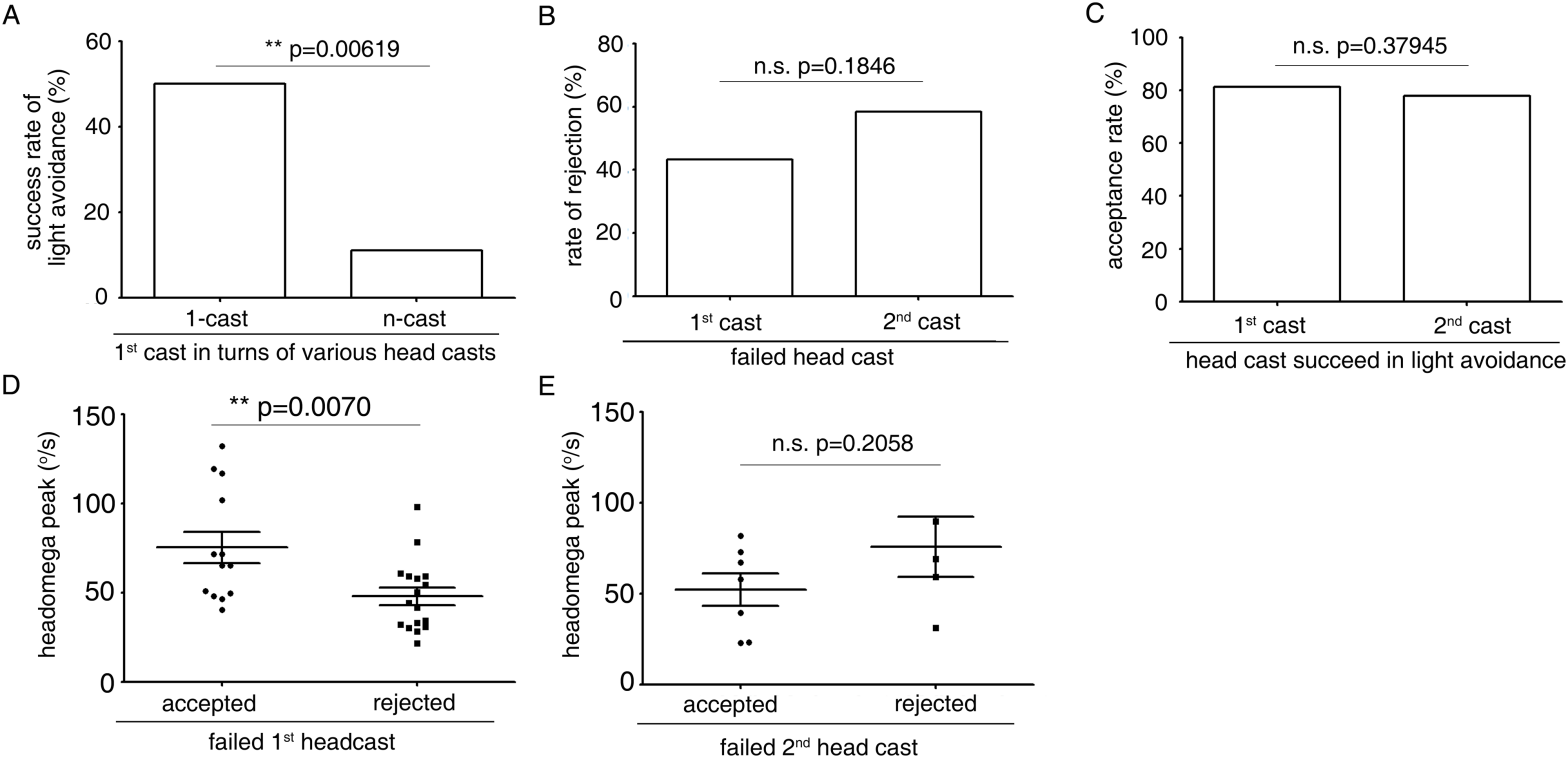
n-cast turns are initiated independently of 1-cast turns. (A) Percentages of successful light avoidance of the first head cast in 1-casts and n-casts in larval fast phototaxis. n = 28 for 1-cast, n = 18 for n-cast. (B) Percentages of larvae that rejected failed first and second head casts. n = 30 for 1^st^ cast, n = 12 for 2^nd^ cast. (C) Rates of acceptance of first and second head casts that were successful in light avoidance. n = 16 for 1^st^ cast, n = 9 for 2^nd^ cast. (D) The magnitudes of accepted first head casts that failed in light avoidance were significantly larger than those that were rejected. The magnitude of head cast was measured by peak value of headomega. n = 13 for accepted, n = 17 for rejected. (E) The headomega magnitudes of accepted second head casts that failed in light avoidance were similar to those of the rejected casts. The magnitude of head cast was measured by peak value of headomega. n = 7 for accepted, n = 5 for rejected. **, *P*<0.01; n.s., not significant; Fisher’s exact test for A, B, C, *t*-test for D and E.

Why do n-cast turns play such an important role in light avoidance? One possible explanation is that if the first head cast fails in light avoidance, the larva performs another head cast to escape from light. Indeed, as shown in Fig 3a, only 11.11% of the first head casts in n-cast turns were successful in light avoidance, whereas for 1-cast turns 50.00% were successful. Thus, only 34.78% of all first head casts were successful (Fig 3A). That is, about 65% of all first head casts failed in light avoidance. Of these failed first head casts, 56.7% were rejected and 43.3% were accepted (Fig 3B). The accepted ones were 1-cast turns; the rejected ones became n-cast turns.

The observation that approximately equal proportions of larvae accepted and rejected the first failed head cast prompted us to reconsider the relationship between 1-cast turns and n-cast turns. As an alternative to the explanation that an n-cast turn is initiated if the first head cast fails in light avoidance, we proposed that a larva might also decide to initiate n-cast at the very beginning. As shown in Fig 3C, if a head cast was successful in light avoidance, the rate of acceptance of this head cast was around 80% (for first head cast, 81.25%; for second head cast, 77.8%; Fig 3C). This means that few further head casts would be initiated after a successful head cast and, therefore, the ratio of rejected successful first head cast in n-casts would be quite low. If the first explanation for initiation of n-cast stands, the failed first head cast in 1-casts and n-casts should not differ in behavioral appearance, because the larva decided on rejection or acceptance of the head cast after it was completed. However, as shown in Fig 3D, the amplitudes of the first failed head cast in 1-casts were significantly larger than in n-casts. We also compared the amplitude of accepted and rejected failed second head casts. The difference was not significant (Fig 3E). This suggests that initiation of head casts after the second might be decided after completion of the second head cast.

Taken together these data suggest that n-cast turns are probably independent of 1-cast turns. Larva might plan to perform an n-cast or a 1-cast turn at the very beginning of turning behavior, irrespective of the result of the first head cast.

### Properties of second head cast were different from those of first head cast in n-cast turns

**Fig 4:**
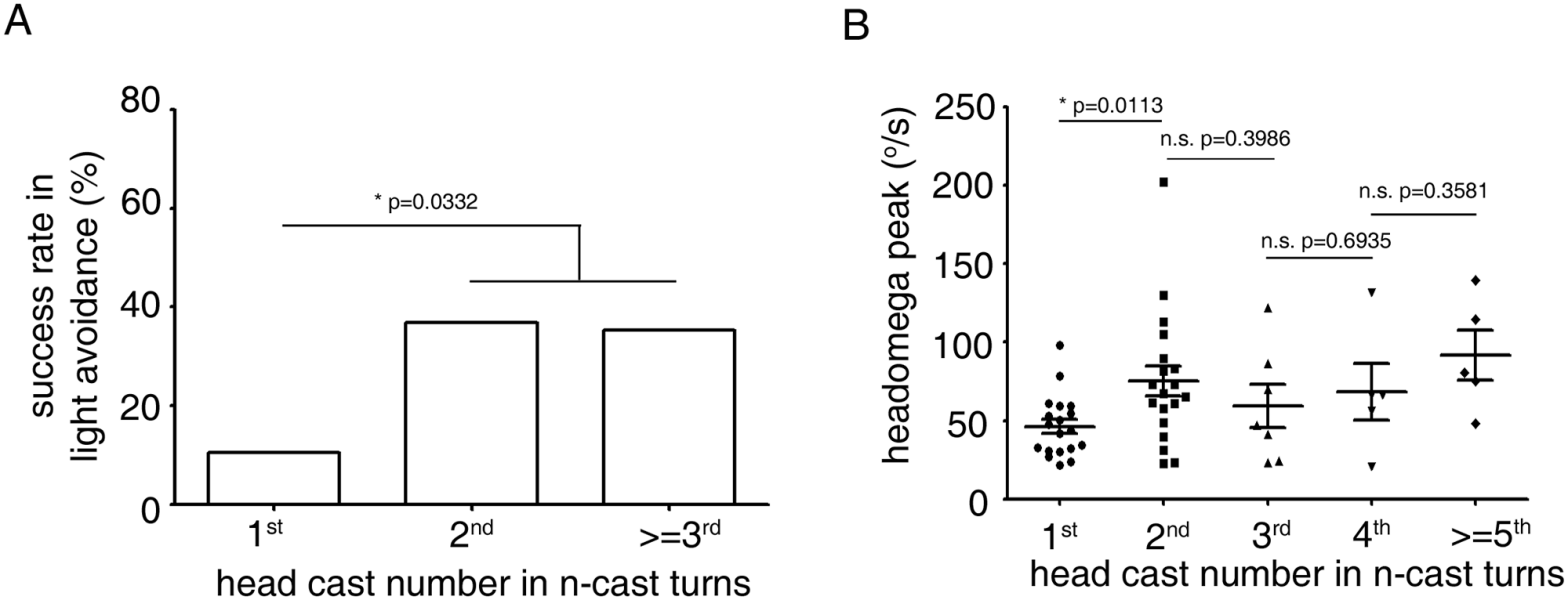
Second head casts are different from first head casts in n-cast turns. (A) Success rates of first-cast, second-cast and n> = 3-casts in n-cast turns in light avoidance. n = 19, 19 and 17 for 1^st^, 2^nd^ and > = 3^rd^ cast.*,*P*<0.05, Fisher’s exact test. (B) Peak headomega values of head casts in n-cast turns. The amplitude of the second head cast is significantly larger than that of the first head cast. n = 19, 19, 7, 5 and 5 for 1^st^, 2^nd^, 3^rd^, 4^th^and > = 5^th^ cast. *,*P*<0.05, n.s. not significant, *t*-tests.

We next asked whether there was a difference between successive head casts in n-cast events. As shown in Fig 4A, the success rate of the second head casts and those after was higher than that of the first head casts, while the success rate of the second head casts was similar to that of the > = 3^rd^ head casts. This means that in n-cast turns, head casts after the second were more effective in light avoidance than the first head cast, although the further head casts were not more effective than the second. We also examined the magnitudes of head casts in n-casts. Interestingly, the magnitude of the second head cast was significantly greater than that of the first head cast (Fig 4B). This suggests that, in an n-cast event, the first head cast may be used only to explore the environment and confirm that one direction was unfavorable, before deciding to execute the larger head casts that follows. After the second head cast, the sizes of head casts did not change significantly, although the trend after the 3^rd^ head cast was to increase gradually (Fig 4B).In n-cast events of more than 5 casts, amplitude of head cast by the 5^th^ or 6^th^ reached levels much higher than those of the previous head cast (S6 Fig). These data suggested that in an n-cast event, larvae first explore the environment with the first head cast and that a turning choice is often made atthe second head cast. If light avoidance continues to be unsuccessful, further multiple head casts are tried until successful avoidance is achieved.

## Discussion

For this study, we developed a light-spot-based phototaxis assay for individual *Drosophila* larvae that typically requires less than one minute to complete, and usually includes a single light entry and exit. The movement of larval body parts can be tracked at high temporal and spatial resolution so that the dynamics of larval phototactic behavior can be analyzed in details. Based on this system, we investigated head-casting behavior, which is considered to be an explorative act preceding a decision by the larva to turn its body to avoid light. We found that upon entering a light region, more than 70% of larvae successfully escaped from the light region. However, after the first head-cast trial, the overall probability of successfully moving the head to a ‘safe place’ (i.e. a dark region) was <40%. Thus in addition to 1-cast turns, turns with multiple head casts play an important role in achieving a high success rate in larval light avoidance.

Light-avoiding turning can be initiated at different time after light stimulation. For head casts that start and end all in light, their contribution to light avoidance is to move larval head closer to light edge so that it is easier for the next head cast to move the head out of light spot. The head casts that start in light but end out of light are the ones that decisively accomplish light avoidance. For head cast starting outside of light spot, those move head further away from light are the decisive factors for light avoidance especially for larvae highly sensitive to light, since they can avoid light before being strongly stimulated. For those head casts that start out of light and end in light, there are two types: the first type of head casts, especially those with large amplitude, appear to be a “wrong” response to light; the second type of head casts, mostly small sized ones, may be tentative since it is often followed by light-to-dark head cast as if the larva dips a little into light to make sure it is light spot and then makes a decisive head cast to leave the light spot. None of these types of head casts seems to be redundant for larval light avoidance.

In our study, we used peaks in headomega to define a head cast event. While headomega is the angular speed of head-midpoint-tail angle, it reflects mainly how fast a larva cast its head, but not the extent that the larva biases its head from the body axis. Generally, large angle head cast results from fast head cast. So headomega can be a sensitive and effective indicator of head cast event. On the other hand, headtheta, a parameter that measures the value of head-midpoint-tail angle, is not as sensitive, especially when a larva withdraws its head to restore body gesture from bended to straight. However, for a larva that can cast its head to large head-midpoint-tail angle at very slow speed, headomega may not be a good choice for defining head cast since peaks in headomega curve will be harder to be accurately detected.

One unexpected but interesting observation was that the amplitudes of first head cast in n-casts and 1-casts were different. This implies that casts after the first may not necessarily result from rejection of the first cast. Or else, the amplitudes of the first head cast in n-casts and 1-casts should be similar. Thus, it seemed that a larva might plan to execute either an n-cast or a 1-cast before it performed the first head cast. The head casts in n-cast turns maybe programmed as a series of casts, like locomotor steps programmed by a CPG (central pattern generator).

In n-cast, the first head cast was generally smaller than second head cast. This might be because some larvae did not directly cast their head to avoid light. In fact, when exposed to elevated light intensity, some larvae usually cast their head toward the direction of light region, as if attempting to confirm the existence of the light spot with respect to its body. A second head cast of larger amplitude was then made to move the head out of the light. This is a reasonable strategy if light stimulation is unfavorable but not extremely dangerous. A test-and-decide strategy helps the larva improve its chances of succeeding in light avoidance.

Although n-casts and 1-casts may be independent behaviors, we could not exclude the possibility that some of them were related. Some n-casts might be initiated because the result of the previous head cast was unfavorable. Conversely, a larva might stop further head casts if the first head cast in a planned n-cast brings its head into darkness. Untangling these seemingly complex relationships depends on identification of the neural basis of 1-casts and n-casts.

In n-cast turns, the casts after the second were quite variable in that the sizes of rejected and accepted second casts that failed in light avoidance were similar (Fig 3b). Thus, in contrast to the putative independence of the first and second casts, casts after the second might still depend on the result of the previous cast. That is, the third cast might result from rejection of the second cast, and so on. As the frequency of higher number casts decreased sharply to negligible levels, their overall importance in light avoidance is small.

In summary, we developed a behavioral paradigm that will facilitate analysis of larval quick light responses as well as future work in identifying neural mechanisms underlying light avoidance behavior. Our observations that larval 1-casts and n-casts might be independently planned and initiated should help finer dissection of avoidance behavior and deepen our understanding of this seemingly simple behavior.

## Conclusions

With the help of a light-spot based phototaxis assay, n-cast turns were found to help improve success rate in larval light avoidance. Judging from the observation that the magnitude of the rejected and accepted first head casts were significantly different, n-cast can be planned in parallel with 1-cast rather than simply being a correction of the first head cast that goes in wrong direction. In n-cast events, the size of the second head cast is larger than the first head cast.

## Supporting information

S1 Fig The set up of light-spot-based larval phototaxis assay. Note that the image was taken in light condition for better visualization.
(TIF)

S2 Fig Relative light intensity inside and outside the light spot. The light intensity along the diameter line of light spot is shown. Light intensity was measured by covering foil sheet witha~1 mm^2^ hole on the photometer detector and moving the detector along the diameter line in 1-mm steps. Arrows point to the positions where the jump in light intensity began and ended.
(TIF)

S3 Fig The flow chart of processing of larval behavioral information and light spot to extract light induced larval behavioral events. (TIF)

S4 Fig The light-spot-based larval phototaxis assay. (Left) The light spot is shown white in a rectangular arena. The smoothed edge of the light spot is shown by a red circular line. (Center) A larva heading toward the light spot at the beginning of a test. (Right) A larva has left the light spot at the end of a test. Arrows point to the track of its head during phototaxis.
(TIF)

S5 Fig Amplitude of head cast with respect to heading angle of larva toward edge of light spot. (A) Absolute peak headomega values of the first head cast with respect to the angle between larval heading and the edge of the light spot at the entry of light spot or initiation of a turn for those did not enter light spot. There is no obvious correlation. (B) Absolute peak headomega values of the second head cast with respect to the angle between the larval heading and the edge of the light spot at the entry of light spot or initiation of a turn for thos did not enter light spot. There is no obvious correlation.
(TIF)

S6 Fig Amplitude of head casts in 6-cast events. Note that by the 5^th^ or the 6^th^ cast, the headomega peaks increases to levels that are much higher than those of previous 1^st^ to 4^th^ head casts.
(TIF)

S1 Video A larva entering and leaving the light spot after multiple head casts. (WMV)

S2 Video A larva showing a long series of head cast when entering light spot. (WMV)
