# Supplementary figures and images for: Turns with multiple and single head cast mediate Drosophila larval light avoidance
Source: PLoS One. 2017 Jul 11;12(7):e0181193. doi: 10.1371/journal.pone.0181193 (PMC5507455; doi:10.1371/journal.pone.0181193)

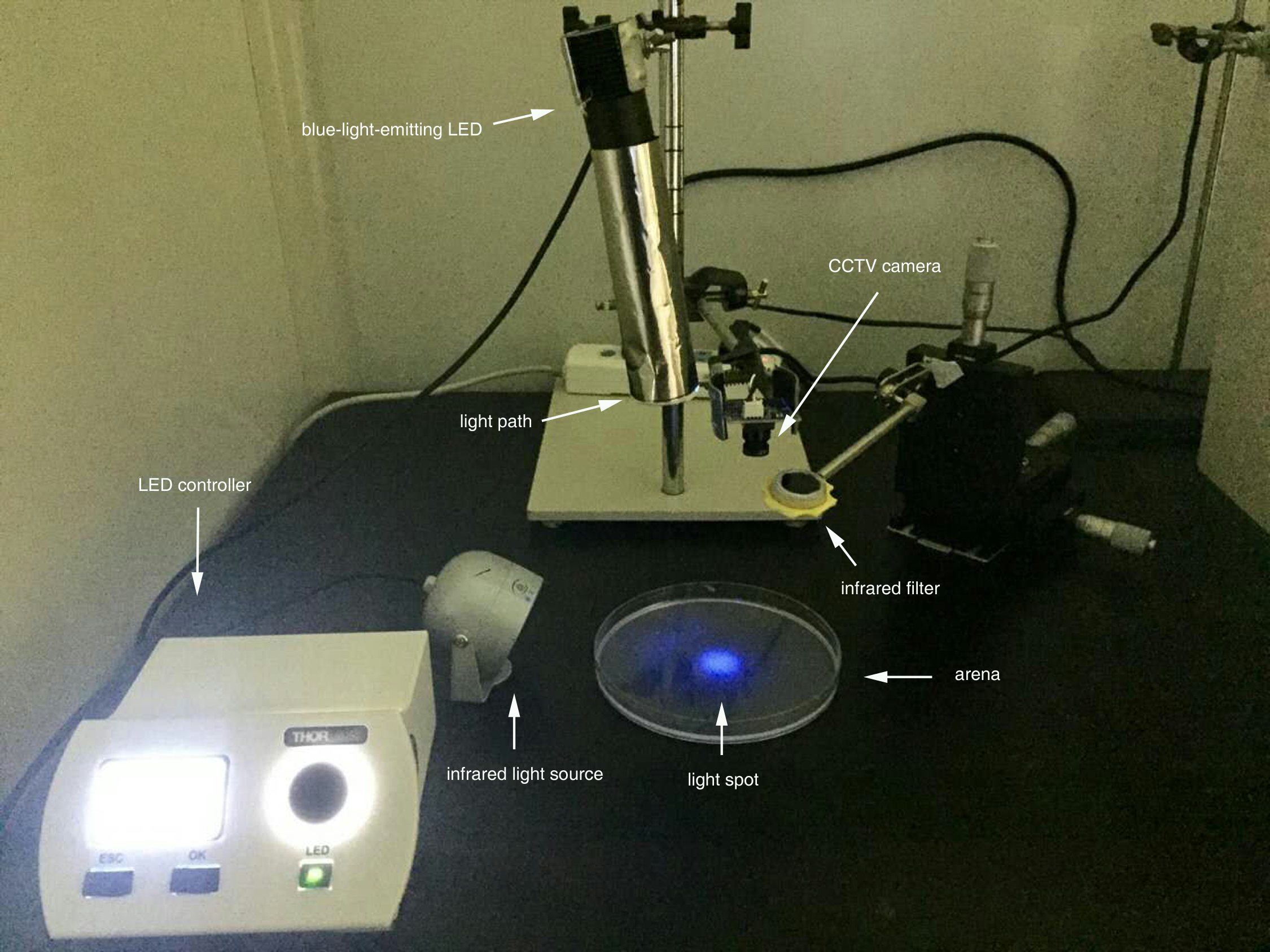

Supplement: S1 Fig — Note that the image was taken in light condition for better visualization. (TIF) [file pone.0181193.s001.tif]

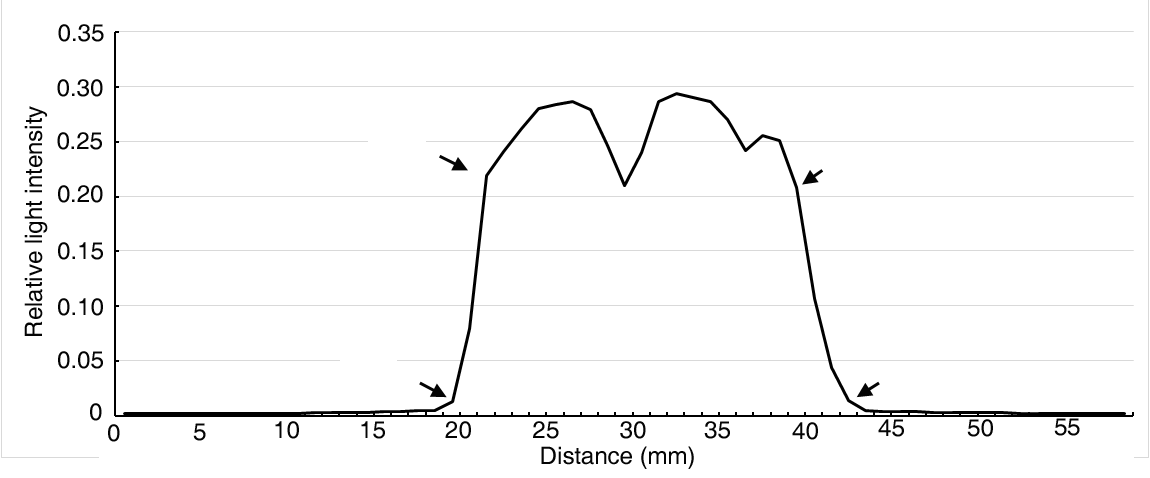

Supplement: S2 Fig — The light intensity along the diameter line of light spot is shown. Light intensity was measured by covering foil sheet witha~1 mm2 hole on the photometer detector and moving the detector along the diameter line in 1-mm steps. Arrows point to the positions where the jump in light intensity began and ended. (TIF) [file pone.0181193.s002.tif]

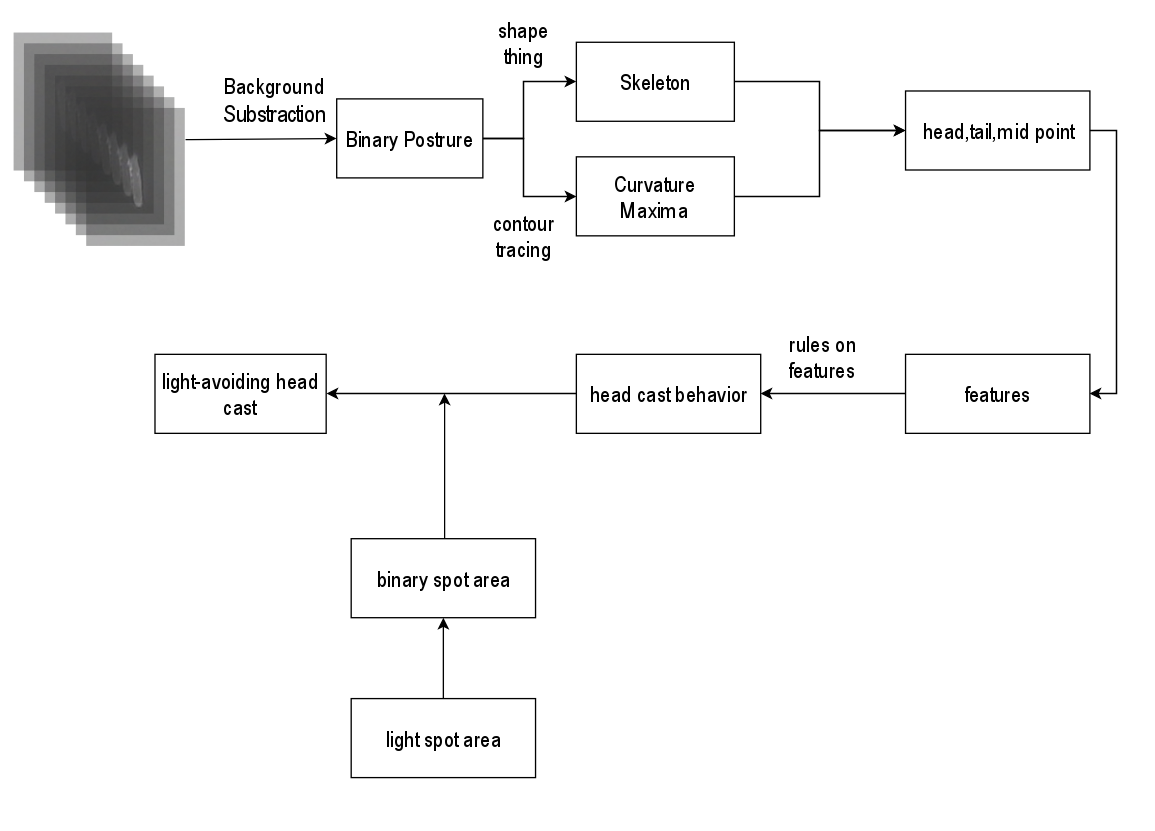

Supplement: S3 Fig — (TIF) [file pone.0181193.s003.tif]

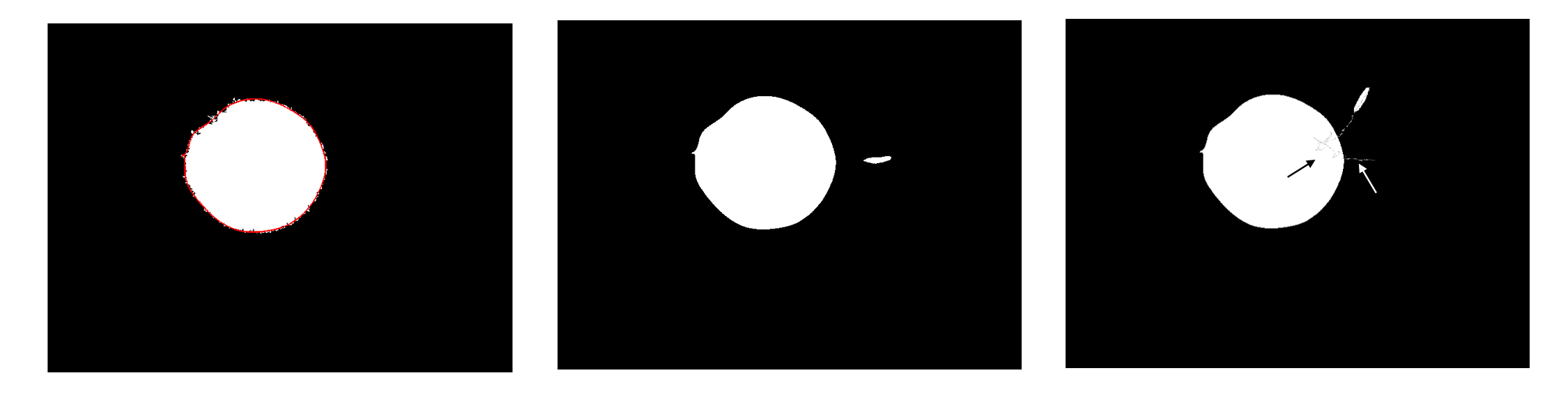

Supplement: S4 Fig — (Left) The light spot is shown white in a rectangular arena. The smoothed edge of the light spot is shown by a red circular line. (Center) A larva heading toward the light spot at the beginning of a test. (Right) A larva has left the light spot at the end of a test. Arrows point to the track of its head during phototaxis. (TIF) [file pone.0181193.s004.tif]

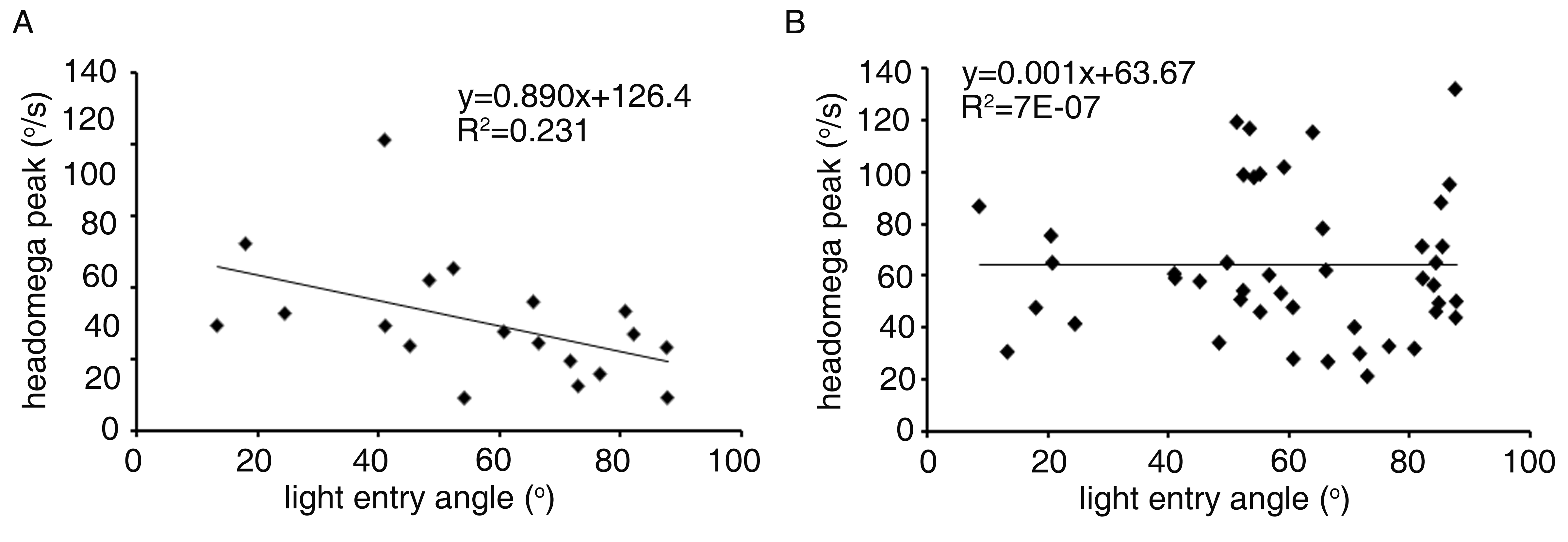

Supplement: S5 Fig — (A) Absolute peak headomega values of the first head cast with respect to the angle between larval heading and the edge of the light spot at the entry of light spot or initiation of a turn for those did not enter light spot. There is no obvious correlation. (B) Absolute peak headomega values of the second head cast with respect to the angle between the larval heading and the edge of the light spot at the entry of light spot or initiation of a turn for thos did not enter light spot. There is no obvious correlation. (TIF) [file pone.0181193.s005.tif]

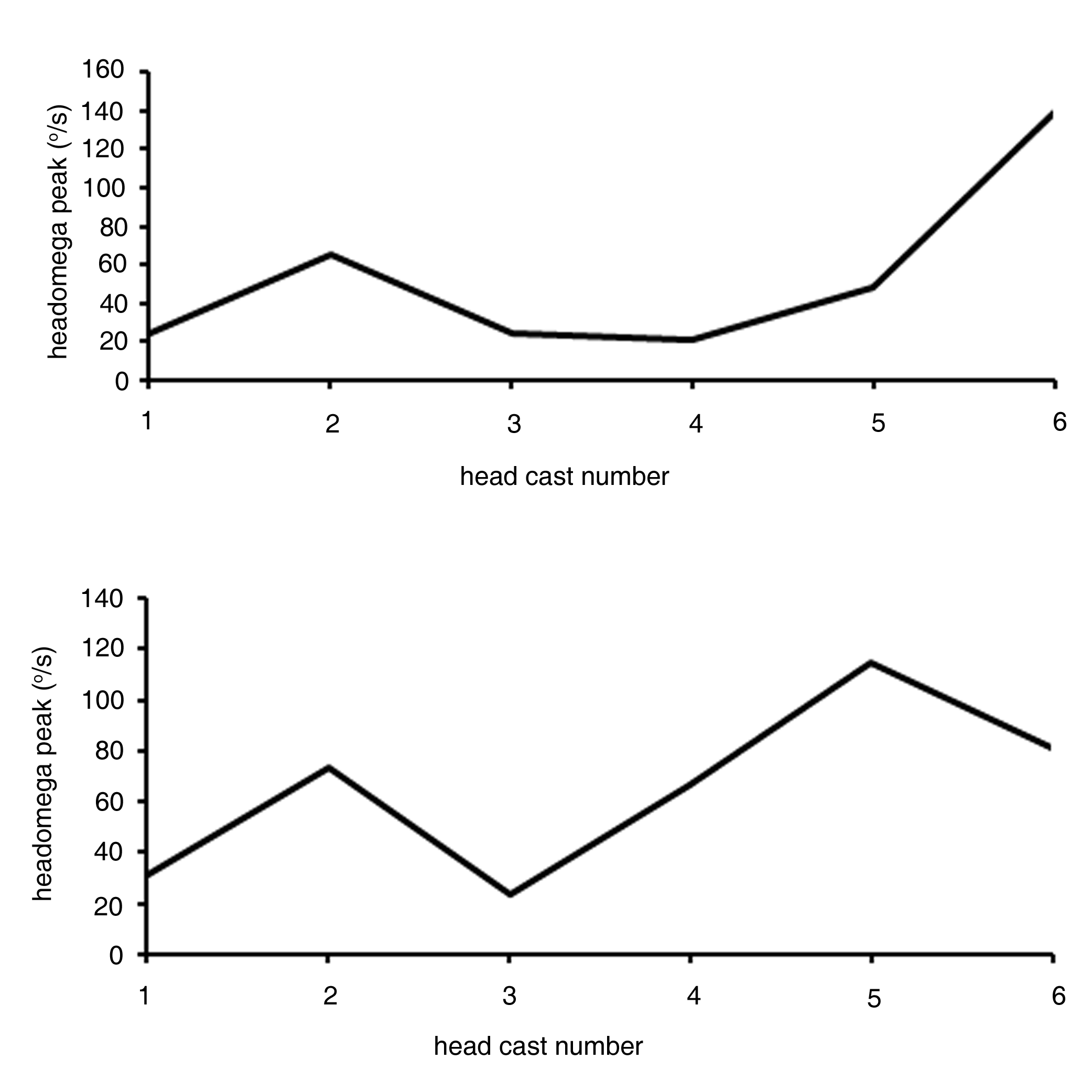

Supplement: S6 Fig — Note that by the 5th or the 6th cast, the headomega peaks increases to levels that are much higher than those of previous 1st to 4th head casts. (TIF) [file pone.0181193.s006.tif]
